# Ultrasound-based population survey for abdominal cystic echinococcosis in Albania: Results from the NDTND project

**DOI:** 10.1371/journal.pntd.0013784

**Published:** 2025-12-02

**Authors:** Tommaso Manciulli, Renata Shkjezi, Valbona Gjoni, Joachim Richter, Laura Lapini, Agnese Colpani, Andrea De Vito, Gian Luca D’Alessandro, Armanda Leka, Argjend Kubolli, Armida Agastra, Gjetjon Gjonaj, Massimo Fabiani, Bruno Zappacosta, Enrico Brunetti, Adriano Casulli

**Affiliations:** 1 Department of Clinical, Surgical, Diagnostic and Pediatric Sciences, University of Pavia, Pavia, Italy; 2 Department of Clinical and Experimental Medicine, University of Florence, Florence, Italy; 3 Cabrini ONLUS Foundation, Sant’Angelo Lodigiano, Italy; 4 Institute of Public Health, Tirana, Albania; 5 Institute of International Health, Charité Centre for Global Health, Charité University Medicine, Corporate Member of Free and Humboldt University and Berlin Institute of Health, Berlin, Germany; 6 Swiss Tropical and Public Health Institute Basel, Basel, Switzerland; 7 Unit of Infectious and Tropical Diseases, Ospedale San Donato, Arezzo, Italy; 8 Department of Medicine, Surgery and Pharmacy, University of Sassari, Sassari, Italy; 9 Department of Pharmacological Sciences, Faculty of Farmacy; Catholic University “Our Lady of Good Counsel”, Tirana, Albania; 10 Unit of Epidemiology, Biostatistics and Mathematical Modelling. Department of Infectious Diseases, Istituto Superiore di Sanità, Rome, Italy; 11 Unit of Infectious and Tropical Diseases, San Matteo Hospital Foundation, Pavia, Italy; 12 WHO Collaborating Centre for the Epidemiology, Detection and Control of Cystic and Alveolar Echinococcosis (One Health), Department of Infectious Diseases, Istituto Superiore di Sanità, Rome, Italy; 13 European Union Reference Laboratory for Parasites (EURL-P; food safety), Department of Infectious Diseases, Istituto Superiore di Sanità, Rome, Italy; Research Center for Hydatid Disease in Iran Kerman University of Medical Sciences, IRAN, ISLAMIC REPUBLIC OF

## Abstract

**Background:**

Albania is considered endemic for cystic echinococcosis (CE). Recent data on prevalence and incidence of CE in Europe suggests that the Balkan region is the epicentre of this neglected parasitic disease in Europe. In Albania, a retrospective study has estimated a mean incidence of 1.49 cases/100,000 people. We aimed to assess the prevalence of CE in eight municipalities across the country.

**Methodology/principal findings:**

We enrolled participants aged >5 years in an ultrasound (US)-based screening for CE in 23 villages in eight municipalities from three prefectures. Participants were enrolled in the study after signing an informed consent and underwent a complete abdominal US. CE cysts were classified according to the WHO-IWGE ultrasound classification. A total of 3,710 participants were included in the study, of whom 2,685 (72.4%) were female. The median age of participants was 55 years (interquartile range, 42–64). Six confirmed CE cases were identified by US with cysts in the liver (1 CE2, 2 CE3a, 1 CE4 and 2 CE5). The crude prevalence of CE detected by US was 0.16% (95% CI 0.06-0.35), with a standardized prevalence of 0.11% (95% CI 0.02-0.21) according to the reference rural population 2023 in Albania. We calculated that the number of individuals who might currently be infected with CE in the rural area of this country was 908 (95% CI 138-1,678).

**Conclusions:**

This was the first population-based US field survey on CE conducted in Albania. Our screening yielded a lower prevalence than expected. However, the presence of active cysts points to ongoing transmission. Our study had several limitations including the use of a convenience sample that limited the attendance of males and children. In the future, surveillance activities for CE should be strengthened in Albania to better characterize the epidemiology of the disease.

## Author summary

The paper presents results from a nationwide ultrasound (US)-based survey for Cystic Echinococcosis (CE) carried out in Albania. CE is endemic in the Balkans and is a public health issue in Albania. However, only data available on disease burden in the country comes from a hospital-based study. In the epidemiological context, US surveys of the general population have been used to estimate disease burden for CE, and to inform policy makers about disease burden. We carried out a survey in in twenty-three villages involving 3,710 subjects, and found a CE prevalence of 0.16%, with standardized estimates ranging from 0.02 to 0.21%. In this study we found both active and transitional echinococcal cysts, which suggests ongoing transmission of disease in the country. Our study shows that CE is present at a non-negligible rate in Albania, with an estimate of 902 (95% CI 138-1,678) individuals who might be infected with CE in rural areas nationwide. As the first US field screening on CE conducted in Albania, our data will be useful to policymakers and inform health agencies on the prevalence of this neglected disease.

## Introduction

Cystic Echinococcosis (CE) is a parasitic disease caused by the cestode *Echinococcus granulosus sensu lato*, present mainly in the rural communities where animal husbandry is practiced, specifically the raising of sheep [1]. Dogs, harbouring the adult worm stage, act as definitive hosts, while ungulates are intermediate hosts where the larval stage (metacestode) is present. Humans are dead-end hosts with cysts in the liver (around 70%), but also in lungs (19%) or uncommon localizations (11%) [2,3]. The current clinical options for CE are drug therapy with benzimidazoles (alone or in combination with other options), percutaneous treatments such as PAIR (Puncture, Aspiration, Injection, Re-Aspiration), surgery (conservative and radical), and “watch-and-wait” (expectant management with ultrasound follow-up). The disease is usually benign with low mortality but can cause morbidity in patients presenting with complicated cysts, or in uncommon localizations, or when mismanaged [2,4]. Most cysts are asymptomatic and around 50% are inactive at diagnosis [5]. The viability of the cyst is described with staging based on the US guidelines of the WHO-Informal Working Group on Echinococcosis (WHI-IWGE) [2]. US has also been used in population-level- studies and is superior to serological surveys for mass screening of populations in endemic areas. Serological tests can yield both false negative results (as they may not detect very young or inactive cysts) and false positive results (due to cross-reactions with other parasitic infections or environmental exposure). A survey utilizing serology for screening would risk: i) missing cases of inactive cysts and ii) misallocating resources due to the need to test serologically positive patients by US, with many patients ultimately negative by US [6,7]. Specifically, serology can miss early (CE1) and inactive (CE4 and CE5) cysts, with underestimation of true positives [8]. Data on abdominal CE from US-based screenings are more reliable as they detect also asymptomatic cysts. Furthermore, ultrasound staging provides information on active transmission [6,9,10]. In the absence of a reliable notification system, epidemiological studies have calculated CE incidence based on hospital discharge records or surgical registry data [11]. However, hospital-based studies miss both outpatients and those with asymptomatic cysts who never seek medical attention [12]. Recent data from Europe points at the Balkan peninsula as the current hotspot of CE transmission in Europe: a recent study showed an increasing incidence at the hospital level from 1997-2020 [13]. Data for Albania from the same study showed a mean incidence of 2.25 cases/100,000 from 1997 to 2017, and of 2.94/100,000 from 2018 to 2020. A study by Luga and colleagues [14] on records from the main surgical center in the country detected 558 cases confirmed by histology between 2009 and 2019, with a mean incidence of 1.49 cases/100,000 people per year. The difference between the two results can be explained by the latter being a single center study, notwithstanding that the center is a national referral center for CE surgery. This is in line with data from other countries in the area [13].

In the 2021–2030 WHO Roadmap on Neglected Tropical Diseases (NTDs), obtaining prevalence data from endemic countries is considered one of the key actions for CE control [15]. As such, the NDTND project’s main objective was the development of new diagnostic tools to improve control of helminthic neglected diseases including cystic and alveolar echinococcosis. Another aim of the NDTND was to conduct population-based US field surveys to gauge CE prevalence in previously uninvestigated settings. This study aimed to: i) generate data on the prevalence of CE in target rural areas of estimated mid-endemicity in Albania and ii) estimate the total number of people that may be affected by CE in rural Albania on a nationwide level.

## Materials and methods

### Ethics statement

The study protocol was approved by the Ethics Committee of the Our Lady of Good Counsel University (approval n 133; Tirana, 12 February 2020).

All participants signed the written informed consent form just before the study participation and were assisted by study collaborators speaking Albanian. For underage participants, a written consent was obtained from the parent or legal guardian nominated by the parent (e.g., a schoolteacher).

**Fig 1:**
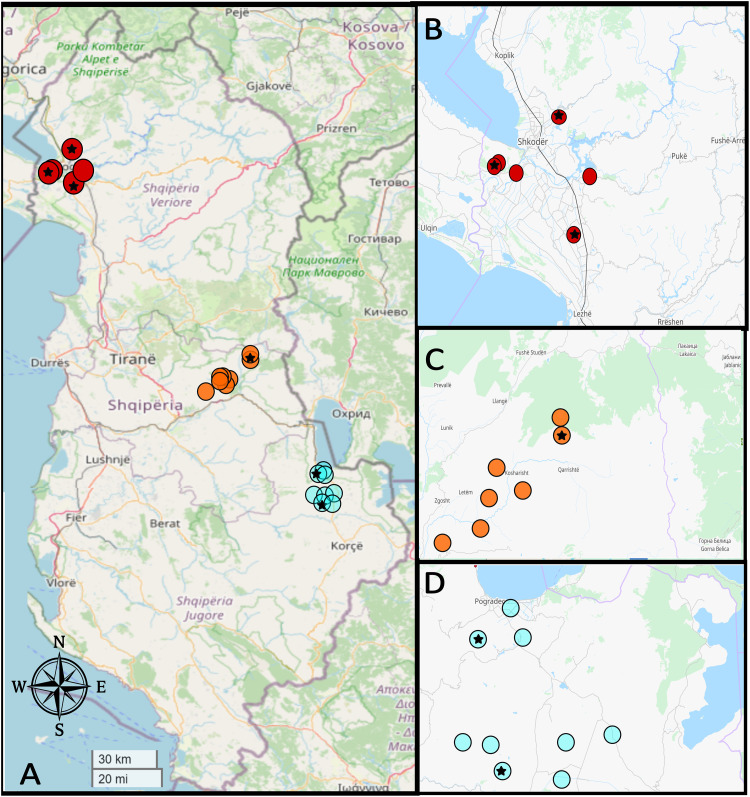
Map showing the distribution of study villages in Albania. Panel A shows a nationwide view of study sites. Red dots = villages in the Shkoder prefecture (detailed view in panel B). Orange dots = villages in the Elbasan prefecture (detailed view in panel C), light blue dots = villages in Korçe prefecture (detailed view in panel D). Dark stars mark the villages where cases of cystic echinococcosis were detected by imaging. Map data was obtained from OpenStreetMap under a CC-BY 2.0 licence (copyright www.openstreetmap.org). Figure Contains information from OpenStreetMap and OpenStreetMap Foundation, which is made available under the Open Database License.

Study regions were selected based on cases recorded by the national reference laboratory for CE at the Albanian Institute of Public Health (IPH; https://www.ishp.gov.al/). The laboratory received all serological positive results, and these were then investigated by contacting the patient to ascertain whether a radiological or anatomopathological diagnosis of CE was made. A further dataset included cases found at a tertiary center in Tirana, Albania. This center (Mother Theresa Hospital) receives patients from all of Albania for CE management, and the dataset was used for a previous publication [14]. Using data from both sources, prefectures with mid-range incidence of CE were selected aiming for a country-wide distribution of study sites (Fig 1). The local team then contacted healthcare providers to gain access to study sites. Sites were then selected based on accessibility and the possibility to ensure adequate logistics. The field coordinator for the project (RS) remained available for further contact with local health authorities. We had initially planned to conduct three separate surveys lasting ten days (one for each region). However, the outbreak of the COVID-19 pandemic forced us to postpone the study from the spring of 2020 to October 2022, and at that later time we could only conduct the study over ten days.

A convenience sample was used, including for ethical reasons all participants who self-presented to screening sites. In the days preceding the surveys, communities were recruited by local healthcare providers who were contacted by RS, explaining the study rationale. A further explanation was carried out when participants signed the informed consent form during the study participation together with study collaborators speaking Albanian. For underage participants, a written consent was obtained from the parent or legal guardian nominated by the parent (e.g., a schoolteacher). A written statement that formal consent was obtained from the parent/guardian. After informed consent, all participants older than five years were offered a US scan of the abdomen carried out through a protocol with standardized views [16]. The scans were carried out by sonographers (TM, LL, GDA, AC, JR) trained in the diagnosis of CE. Doubtful cases were discussed until a consensus on CE diagnosis was reached. All operators had a minimum of one year experience with US and specific training in CE diagnosis and staging. Four US machines were used: one Sonosite M-Turbo (Fujifilm, Japan), one Mindray Z6 and one Mindray M5 (Mindray, China) and one Esaote Sigma handheld ultrasound device (Esaote, Italy). After the US exam, a standard case report form was used for data acquisition and reporting results to participants. Local physicians and three health professionals with training in the management of CE were present during examinations to explain results to participants, including accidental findings. All CE cases were issued recommendations based on the WHO-IWGE Expert Consensus on the clinical management of echinococcosis [2] and participants were offered the possibility to be referred to the public health system for appropriate management. Participants with incidental findings were issued a recommendation to be discussed with local health professionals for further management, if needed.

We calculated the population by sex and age-group living in rural Albania by re-scaling the 2023 census data (INSTAT. Albania population and housing census 2023. Available at: https://www.instat.gov.al/media/13615/cens-i-popullsise-2023.pdf; World Bank. Rural population) according to the overall proportion of the resident population living in urban areas as reported by the World Bank for the same year (35.4%) (World Bank. Rural population - % of total population - Albania. Available at: https://data.worldbank.org/indicator/SP.RUR.TOTL.ZS?locations=AL). The demographic characteristics of the study sample and the 2023 estimated national rural population were described through counts and percentages. We estimated the overall and prefecture-specific crude CE prevalence, as well as prevalence rates adjusted through direct standardisation by sex and age-group using the 2023 country’s rural population as reference. The prevalence in the rural population of Albania was estimated multiplying the adjusted prevalence by the 2023 rural population size. Finally, we estimated the sex and age-group specific CE prevalence and evaluated the association of demographic variables with CE infection through a multivariable logistic model including randomization to account for clustering at the village level. The adjusted odds ratios (OR) were used to describe the strength of these associations. All estimates were presented with 95% confidence Intervals (CI). The analysis was performed using Stata 16.1 (StataCorp, College Station, Texas, USA).

## Results

A total of 23 villages in eight municipalities from three prefectures were visited by the team. Screening site distribution is shown in Fig 1.

A total of 3,710 people were screened by US, of which 1,257 (33.9%) from Elbasan, 1,254 (33.8%) from Korçë and 1,199 (32.3%) from Shkodër. In total, 2,685 (72.4%) participants were female and 1,025 were male (27.6%) (Table 1). The median age of the population screened was 55 years (interquartile range, 42–64) of which 804 (21.7%) people were between 5–39 years, 1,485 (40.0%) between 40–59 years and 1,421 (38.3%) over 60 years (Table 1).

**Table 1:** Comparison between the number of participants in the study, divided by sex and age class, and the national reference population in Albania according to the latest national Albanian Census (2023).

Sample							Reference rural population, 2023					
Group	Women		Men		Total		Women		Men		Total	
Age class (years)	n	%	n	%	n	%	n	%	n	%	n	%
**5-39**	591	22	213	20.8	804	10.8	173,200	42.3	179,929	44.9	353,129	43.6
**40-59**	1,188	44.3	297	29	1,485	40	115,250	28.1	106,028	26.5	221,278	27.3
**≥60**	906	33.7	515	50.2	1,421	38.3	121,437	29.6	114,825	28.7	236,262	29.1
**Total**	2,685	100	1,025	100	3,710	100	409,887	100	400,782	100	810,669	100

A total of six CE cases were detected out of 3,710 eligible people with a crude prevalence of 0.16% (95% CI 0.06-0.35) and a standardized prevalence of 0.11% (95% CI 0.02-0.21) according to the reference rural population 2023 in Albania (Table 2).

**Table 2:** Prevalence of abdominal cystic echinococcosis estimates for the three involved prefectures in Albania. *Directly standardised by age and sex, using the 2023 rural population.

Prefecture	Cases/participants	Crude prevalence (95% CI)	Standardised prevalence* (95% CI)
**Elbasan**	1/1,257	0.08 (0.00-0.44)	0.09 (0.00-0.26)
**Korçë**	2/1,254	0.16 (0.02-0.57)	0.14 (0.00-0.37)
**Shkodër**	3/1,199	0.25 (0.05-0.73)	0.14 (0.00-0.31)

One CE2 cyst (active), two CE3a cysts (transitional), one CE4 cyst and two CE5 cysts (inactive) were detected. In particular, one CE case was detected in Elbasan, two in Korçë and three in Shkodër. All cases but one were newly diagnosed. The known case was diagnosed as a CE3a cyst and was undergoing treatment with albendazole (ABZ) at the time of the screening. Four patients were female, two were males (Table 3). Two cases were detected within the age class 40–59 years and four over 60 years. No statistically significant association was found in a multivariable analysis between CE and sex (p-value 0.854) or age (p-value 0.361) (Table 3). Comparison between age classes and sex did not show a statistically significant difference.

**Table 3:** Abdominal Cystic echinococcosis by imaging in participants by class and probability estimates in our study. *Directly standardised by age and sex, using the 2023 rural population. NE, not estimable. **Odds ratios were adjusted for sex and age and derived from a multilevel logistic model including the survey village as random effect. The odds ratio for age indicates the change of odds per 10-year increase in age.

Demographics	cases/participants (%)	Standardised prevalence* (95% CI)	Odds Ratio** (95% CI)	p-value
**Age 5–39 years**	0/804 (0.00)	0.00 (NE)	1.30 (0.74-2.28)	0.361
**Age 40–59 years**	2/1,485 (0.13)	0.09 (0.00-0.21)		
**Age ≥ 60 years**	4/1,421 (0.28)	0.30 (0.00-0.61)		
**Female**	4/2,685 (0.15)	0.11 (0.00-0.22)	1	0.854
**Male**	2/1,025 (0.20)	0.11 (0.00-0.26)	1.17 (0.21-6.57)	

Using the overall age/sex standardised prevalence estimate and the reference rural population size of Albania in 2023, we calculated that the number of individuals who might currently be infected with CE in the rural area of this country was 908 (95% CI 138-1,678).

Five participants (0.13%) reported a history of surgical treatment for CE when screened. Of these, three participants (0.08%) reported a history of lung CE without abdominal localizations, whereas two participants (0.05%) had a history of liver CE treated by surgery. None of the participants recalled ABZ intake after surgical management, although data on ABZ availability in Albania at the time of treatment was not available.

## Discussion

This was the first US-based field survey on CE conducted in Albania. The country does not have a notification system for CE [14] and reliable information on disease prevalence is needed. Collecting data on CE prevalence is included in the key actions endorsed by the WHO 2021–2030 roadmap for NTDs [15]. The largest epidemiological study conducted for research purposes examined abdominal CE prevalences in two neighbouring countries (Romania and Bulgaria) and found prevalences of 0.41% in both countries [6]. Authors found that commonly used epidemiological indicators (e.g., incidence rates derived from hospital discharge records) systematically underestimate disease burden. The lower prevalence found in our study could not be explained by methodological differences since authors from the HERACLES study on cystic echinococcosis also used provinces with intermediate incidences derived from several data sources to conduct US-based screenings [6]. The prevalence found in Albania is closer to the one found in a survey we carried out in southern Italy [17]. However, only one inactive case was found during that survey. In this study we found active and transitional cysts that suggest ongoing transmission of disease, although it is impossible to state how recent those cysts were as growth rate and time to inactivation vary widely [18], and no cysts were present in children. The presence of early (i.e., CE1) cysts in children has been used as a marker of recent disease transmission to humans since no cyst can be older than the host [9,16]. Overall, we saw a low number of children, likely due to the use of a convenience sample. However, children have consistently shown a lower prevalence of CE during US-based surveys, as the probability of infection in a person living in an endemic area is directly proportional to age and children are expected to have a lower burden of disease [5,10,18,19]. Participants were mostly women. This could be explained by men’s inability to attend due to work commitments, a common occurrence in this kind of study [6,17,19–22]. In our assessment we did not find a significant difference in abdominal CE prevalence when comparing men and women, a finding concordant with the HERACLES study [6,23]. Patients who reported a history of CE should be considered with caution as we were not able to review imaging studies proving CE and patients with a history of surgery for lung CE were included, but lung cysts are not detectable with ultrasound unless located near the pleura. Our estimates for the number of abdominal CE patients in the country suffer from the relatively low sample size, as shown by the wide confidence interval. Nevertheless, our data indicate that CE may represent a significant public health problem in Albania. This is especially true if we consider that most cases of abdominal CE cases are managed with surgery [14] and that previous surveys in comparable settings have shown scarce adherence to expert recommendations on stage-specific approaches and perioperative use of ABZ [21,24].

Our study had several methodological limitations, most of which are common to other US-based population surveys for CE: 1) we used data derived from hospital records of two institutions and cannot exclude a selection bias even if the institutions are national referral centers; 2) the use of a convenience sample comes with a selection bias in our target population. Overcoming this would require a commitment of time and resources that was beyond our budget. Half the cases of CE by imaging have been detected in a single prefecture, in the absence of differences in surgical incidences between prefectures. This could suggest the presence of transmission hotspots, in accordance with results by Paternoster and colleagues who found a clustered pattern of CE incidence in Kyrgyzstan [25]. To conclude, our study represents a first assessment of the prevalence of abdominal CE in Albania. Our results confirm that the disease is present in the country and findings from this study should be used to support the planning of surveillance programmes in Albania according to the WHO 2021–2030 roadmap for neglected tropical diseases.
